# Biosorption of Cr(VI) Using Cellulose Nanocrystals Isolated from the Waterless Pulping of Waste Cotton Cloths with Supercritical CO_2_: Isothermal, Kinetics, and Thermodynamics Studies

**DOI:** 10.3390/polym14050887

**Published:** 2022-02-23

**Authors:** Siti Hajar Mohamed, Md. Sohrab Hossain, Mohamad Haafiz Mohamad Kassim, Venugopal Balakrishnan, Mohamed A. Habila, Azham Zulkharnain, Muzafar Zulkifli, Ahmad Naim Ahmad Yahaya

**Affiliations:** 1 School of Industrial Technology, Universiti Sains Malaysia, Gelugor, Penang 11800, Malaysia; sitihajarmohamed95@gmail.com (S.H.M.); mhaafiz@usm.my (M.H.M.K.); 2 Institute for Research in Molecular Medicine (INFORMM), Universiti Sains Malaysia, Gelugor, Penang 11800, Malaysia; venugopal@usm.my; 3 Department of Chemistry, College of Science, King Saud University, P.O. Box 2455, Riyadh 11451, Saudi Arabia; mahabila@ksu.edu.sa; 4 Department of Bioscience and Engineering, Shibaura Institute of Technology, College of Systems Engineering and Science, 307 Fukasaku, Minuma-ku, Saitama 337-8570, Japan; azham@shibaura-it.ac.jp; 5 Institute of Chemical and Bio-Engineering Technology, Universiti Kuala Lumpur Malaysian, Alor Gajah, Melaka 78000, Malaysia; muzafar@unikl.edu.my

**Keywords:** waste cotton cloths, cellulose nanocrystals, solid waste management, supercritical CO_2_, heavy metals adsorption, sustainability

## Abstract

In the present study, supercritical carbon dioxide (scCO_2_) was utilized as a waterless pulping for the isolation of cellulose nanocrystals (CNCs) from waste cotton cloths (WCCs). The isolation of CNCs from the scCO_2_-treated WCCs’ fiber was carried out using sulphuric acid hydrolysis. The morphological and physicochemical properties analyses showed that the CNCs isolated from the WCCs had a rod-like structure, porous surface, were crystalline, and had a length of 100.03 ± 1.15 nm and a width of 7.92 ± 0.53 nm. Moreover, CNCs isolated from WCCs had a large specific surface area and a negative surface area with uniform nano-size particles. The CNCs isolated from WCCs were utilized as an adsorbent for the hexavalent chromium [Cr(VI)] removal from aqueous solution with varying parameters, such as treatment time, adsorbent doses, pH, and temperature. It was found that the CNCs isolated from the WCCs were a bio-sorbent for the Cr(VI) removal. The maximum Cr(VI) removal was determined to be 96.97% at pH 2, 1.5 g/L of adsorbent doses, the temperature of 60 °C, and the treatment time of 30 min. The adsorption behavior of CNCs for Cr(VI) removal was determined using isothermal, kinetics, and thermodynamics properties analyses. The findings of the present study revealed that CNCs isolated from the WCCs could be utilized as a bio-sorbent for Cr(VI) removal.

## 1. Introduction

There is a growing attentiveness to the sustainable utilization of WCCs to reduce carbon footprints and environmental pollutions [1]. The rise of fast-fashion demand in textile industries has increased cotton cloth production, which has enhanced the generation of WCCs. A large number of WCCs are generated every year. The most preferred disposal method of WCCs is open dumping in landfill sites, along with other domestic and municipal wastes [1,2,3]. This inappropriate disposal practice leads to the demolition of valuable cellulosic materials and poses severe environmental pollutions. Wherein, the anaerobic decomposition of organic materials in WCCs in landfill sites causes greenhouse gas (GHGs) emissions such as methane (CH_4_) and carbon dioxide (CO_2_) [4,5]. Moreover, the presence of chemical dyes in WCCs may increase the acidity of soil and pollute groundwater. However, the WCCs are rich in cellulose, which can be recycled and reused to produce value-added products [5]. 

Cellulose nanocrystals (CNCs) are the crystalline region of cellulose, isolated from cellulose pulp fiber using acid hydrolysis by removing the amorphous region of cellulose while leaving the only crystalline region of cellulose [1,6]. CNCs exhibit as elongated and cylindrical rod of crystalline cellulose with a width of 2–70 nm, length of 100–600 nm, and crystallinity index of 75–88% [6,7,8]. The distinct properties of CNCs, such as its light weight, its distinctive optical properties, its abundant availability, its high surface area, its excellent thermal and mechanical strength, its biodegradability, and its environmental friendliness, make CNCs an encouraging raw material for many advanced implementations [8]. In recent years, CNCs have been used in many advanced implementations, such as packaging materials [9], filler or reinforcement in composite materials [10], electronics [11], biomedical engineering [12], and bio-sorbents [13]. 

The isolation of CNCs from WCCs requires various processing steps, including delignification of lignocellulosic biomass for the extraction of cellulose and conversion of cellulose to CNCs [1,6,9]. The existing process for the extraction of cellulose from the WCCs is the combination of alkaline pulping and bleaching processes. However, the extraction of cellulose using conventional pulping and bleaching requires toxic chemicals and consumes a massive amount of freshwater, resulting in the generation of toxic effluent [1,6]. Therefore, environmentalists and scientists are keen to define alternative technologies for minimizing the toxic effluent generation from the cellulose extraction of various lignocellulosic biomass. Supercritical CO_2_ (scCO_2_), as a waterless extraction technology, has been extensively used in the extraction of lipids [14,15], bioactive compounds [16], and antioxidants [17]. The main advantages of scCO_2_ extraction technology are that this technology is a low-temperature extraction technology, it does not generate any waste or toxic effluents, and it does not require any purification processes for the final product [14,17]. Moreover, fluid CO_2_ is cheap, is widely available, has a moderate critical pressure (7.4 MPa), has a low critical temperature (31.1 °C), is non-toxic, is non-flammable, and is environmentally friendly [18]. However, scCO_2_ extraction technology has been utilized in the delignification of lignocellulosic biomass for the extraction of cellulose. Pasquini et al. [19] utilized scCO_2_ for pulping sugar cane bagasse with the addition of a co-solvent (1-butanol/water). Vedoya et al. [20] found that scCO_2_ pulping influences the cellulose yield and hemicellulose removal with a shorter reaction time at a lower temperature than the hydrothermal pulping process. 

The most used method for the CNCs’ isolation from a cellulosic source is the acid hydrolysis process [1,21]. Sulfuric acid and hydrochloric acid are the main mineral acids used in acid hydrolysis processes for the CNCs’ isolation [21,22]. However, CNCs isolated with HCl hydrolysis have a number of limitations, including poor dispersion ability, weak oxidizing ability, and low thermal degradation [1,22]. Conversely, the CNCs isolated with the H_2_SO_4_ hydrolysis provide better mechanical and thermal stability due to stable cellulose suspension and the fact that they contain the sulfate group on the surface of the crystallites [6,21,23]. Water pollution and heavy metal pollution issues raise severe environmental concerns. Hexavalent chromium Cr(VI) is commonly discharged from leather tanning, wood, textile dyeing, metal processing industries, and electroplating [24,25]. Cr(VI) is a highly toxic, carcinogenic, teratogenic and mutagenic element [25]. Therefore, the discharge of Cr(VI) in the environment can be detrimental to human health and the environment. Hence, in order to protect the environment, Cr(VI) must be efficiently eliminated from industrial effluent before it is discharge in any body of water.

Several treatment methods have been employed for Cr(VI) removal in wastewater treatment, including ion exchange, membrane filtration, chemical precipitation, ion exchange, and adsorption [24,26]. Of these, ion exchange and membrane filtration are viewed as the most effective treatment methods for the removal of Cr(VI) from industrial effluent. However, these processes are not commercially applicable for the removal of Cr(VI) from the industrial effluent due to their high operating cost [27,28]. Conversely, adsorption is a simple, versatile, and commercially viable process for the removal of Cr(VI) from the industrial effluent [27]. Over the years, various biobased materials are being used as an adsorbent to remove Cr(VI) from industrial effluent [27,29]. Studies reported that adsorption using CNCs is considered a promising method for Cr(VI) removal because of its easy handling, high efficiency, high adsorption rate, reusability, and economic feasibility [24,29]. The prime objective of this study was to evaluate Cr(VI) removal performance using isolated CNCs from WCCs as an adsorbent. To this end, the cellulose was extracted from the WCCs using waterless scCO_2_ with the addition of ethanol (EtOH) as a co-solvent. CNCs were isolated from the scCO_2_ extracted cellulose using the sulphuric acid hydrolysis method. Subsequently, the isolated CNCs were utilized as an adsorbent for Cr(VI) removal with varying pH and adsorbent doses’ adsorption time and temperature. Finally, isothermal, the kinetic and thermodynamic behavior of Cr(VI) removal using isolated CNCs was determined. The findings of this study will be useful to determine the sustainable utilization of WCCs with minimizing municipal waste disposed of in landfill and environmental pollution. 

## 2. Materials and Methods

### 2.1. Sample Collection and Preparation

WCCs were obtained from Pulau Burung Landfill Site, Penang, Malaysia. After collection, the WCCs were cleaned and sterilized using scCO_2_ at a temperature of 60 °C and a pressure of 20 MPa for a treatment time of 1 h [30]. The sterilized WCCs were then shortened into small pieces (approx. 2 cm × 2 cm) and stored in a refrigerator at 4 °C.

### 2.2. Cellulose Nanocrystals Isolation from WCCs

The sterilized WCCs underwent delignification with scCO_2_ pulping at a temperature of 60 °C, an scCO_2_ pressure of 17 MPa, a co-solvent of 15 wt.% ethanol, and a treatment time of 90 min. The CNCs were isolated from scCO_2_ pulped fibre using 64 wt.% H_2_SO_4_ solution at a temperature of 45 °C and 1:10 (g/mL) of solid to liquid ratio for a 1 h treatment time to enhance the crystallinity of cellulose by hydrolyzing the amorphous cellulose. To stop the reaction, the suspension was then diluted with cool deionized water (about twice the initial acid volume). Subsequently, the suspension was centrifuged at 10,000 rpm for 10 min and washed with deionized water repetitively to eliminate the excess acid. Subsequently, the suspension was placed in a cellulose membrane dialysis tube to remove the excess sulfate ions by altering deionized water until a pH of 7 was attained. The suspension (in gel form) was homogenized using a homogenizer for 5 min at a speed of 5 m/s. Subsequently, the suspension was further homogenized using ultrasonication in an ice water bath at 80% amplitude and a frequency of 0.3 cps for 15 min. The suspension was then dried using a freeze-drying process at −50 °C, 0.5 torrs of vacuum pressure, and a drying time of 3 days. After 3 days of the freeze-drying process, a dry CNCs powder was collected for characterization.

### 2.3. Adsorption of Cr(VI) Using CNCs

The Cr(VI) adsorption was carried out using the isolated CNCs as the adsorbent with varying pH (pH 1–pH 8), adsorbent doses (0.5–2.0 g/L), treatment time (15–90 min), and temperature (ambient −80 °C). Certain numbers of CNCs were placed into an Erlenmeyer flasks, which contained 50 mL of Cr(VI) solution. Later, the mixture was mixed with an incubator shaker under vigorous agitation at 150 rpm. The concentrated H_2_SO_4_ and NaOH solutions were used to adjust the pH of the aqueous solution. After adsorption, the CNCs were separated using filter paper, and the Cr(VI) concentration in the effluent was anticipated by atomic absorption spectrophotometry (Shimadzu, AA-7000, Kyoto, Japan). The percentage removal of Cr(VI) was evaluated as follows:

(1) $$Removal=\frac{C_{i}-C_{t}}{C_{i}}\times 100\;\;$$

where *C_t_* and *C_i_* are the Cr(VI) concentration(mg/L) at time *t* and the initial Cr(VI) concentration (mg/L). The Cr(VI) removal capacity at equilibrium (*q_e_*) was determined using Equation (2).

(2) $$q_{e}=\frac{C_{i}-C_{e}}{D}\times V\;$$

where *V* represents the volume (L) of the aqueous solution, *C_e_* denotes the Cr(VI) concentration at equilibrium (mg/L), and *D* is the amount of CNCs (mg). Triplicate measurements were conducted, and the results were expressed as means ± standard error.

### 2.4. Adsorption Isotherm

The experiments were carried out with varying CNCs adsorbent dosage (0.25–2.0 g/L) as a function of the treatment time (10–60 min) at pH 2, an initial Cr(VI) concentration of 100 ppm, and at ambient temperature (28 ± 1 °C). The experimental data were fitted with the predicted values from the Freundlich and Langmuir isotherm models. The best-fitted model was predicted by the linear regression model to elucidate the adsorption behavior of CNCs for Cr(VI) removal. The Freundlich and Langmuir isotherm model can be expressed in the ways shown in Equations (3) and (4), respectively.

(3) $$q_{e}=K_{f}C_{e}^{\frac{1}{n}}\;$$

(4) $$q_{e}=\frac{abC_{e}}{1+aC_{e}}\;$$

where *K_f_*, *n*, *a*, and *b* are the Freundlich affinity coefficient (L/mg), Freundlich exponential constant, Langmuir constant, and the optimal adsorption value for Cr(VI) removal using CNCs as an adsorbent. The linear form of the Freundlich and Langmuir isotherm model can be expressed in the ways shown in Equations (5) and (6), respectively.

(5) $$\log q_{e}=\log K_{f}+\frac{1}{n}\log C_{e}\;\;$$

(6) $$\frac{1}{q_{e}}=\frac{1}{abC_{e}}+\frac{1}{b}\;$$

### 2.5. Kinetics and Thermodynamics Modelling

To assess the kinetic and thermodynamic behavior of the Cr(VI) removal using CNCs, the experiments were performed with temperatures varying from 28 °C to 80 °C as a function of the treatment time from 10 min to 60 min at initial [Cr(VI)] of 100 ppm, pH 2, and a CNCs dosage of 1.5 g/L. The kinetic behavior of the Cr(VI) removal using CNCs was determined with the pseudo-first kinetic model and pseudo-second-order kinetic model. The pseudo-first kinetic model and pseudo-second-order kinetic model equations are presented in Equations (7) and (8), respectively.

(7) $$\ln(q_{e}-q_{t})=\ln q_{e}-k_{1}t\;$$

(8) $$\frac{t}{q_{t}}=\frac{1}{k_{2}q_{e}^{2}}+\frac{t}{q_{e}}\;$$

where *q_e_* and *q_t_* represent the maximum Cr(VI) adsorption and Cr(VI) adsorption at time *t* (min), respectively, *k*_1_ (1/min) and *k*_2_ (mg/mg.min) denote the pseudo-first-order rate constant and pseudo-second-order rate constant. The thermodynamics variables including enthalpy changes (Δ*H°*), Gibbs free energy changes (Δ*G°*), and entropy changes (Δ*S°*) were determined to predict the thermodynamic behavior of Cr(VI) removal using CNCs as an adsorbent. The thermodynamics parameters were analyzed using the following equations:

(9) $$\Delta G{}^{\circ}=-RT\ln K{{}^{\circ}}^{}\;$$

(10) ΔG°=ΔH°−TΔS° 

(11) $$\ln K{}^{\circ}=\frac{\Delta S{}^{\circ}}{R}-\frac{\Delta H{}^{\circ}}{RT}\;$$

where *R* (8.314 × 10^−3^ kJ/mol.K) denotes the universal gas constant, *K°* denotes the Gibbs constant, and *T* represents the Kelvin temperature. The *K°* value was predicted from the ratio of *q_e_* to *C_e_*. Subsequently, Δ*H°* and Δ*S°* were determined by analyzing the slope and intercept of the linear plot of ln *K°* versus 1/*T*, respectively. 

### 2.6. Characterization

Field emission scanning electron microscopy (FE-SEM) was utilized to determine the surface morphology of the WCCs’ fiber, scCO_2_ pulped fiber, and the CNCs isolated at an accelerating voltage of 5 kV. Moreover, the energy-filtered transmission electron microscopy (TEM-Libra 120, Carl Zeiss, Oberkochen, Germany) was utilized to capture the image and dimensions of CNCs isolated from CNCs at an accelerating voltage of 80 kV. A small droplet of 0.1 wt.% CNCs suspension was deposited on the copper grid, followed by staining using a negative stain of 2 wt.% uranyl acetate solution. Subsequently, the length and diameter of CNCs particles were dignified with an image analysis software. Atomic force microscopy (AFM-Bruker Multimode 8, Billerica, MA, USA) was also used to determine the surface morphology of CNCs at a scan area of 1 µm × 1 µm.

Fourier transform infrared spectroscopy (FTIR) equipped with an attenuated total reflectance (ATR) accessory was utilized to determine the functional groups present in the WCCs’ fiber, scCO_2_ pulped fiber, and isolated CNCs at a scanning speed of 20 mm/s, a spectral resolution of 4 cm^−1^, a wavenumber of 600–4000 cm^−1^, via 32 scans. Zeta potential (ζ) analysis was accompanied to define the surface charge of particles, which can be used to evaluate colloidal dispersion stability. The ZetaSizer Nano-ZS equipment (Malvern, Worcestershire, Worcester, UK) was used to determine the Zeta potential of CNCs at pH 7, a detecting angle of 173°, a wavelength of 633 nm, a viscosity of 0.8872 cP, and a water refractive index of1.33. The size distribution and particle size of CNCs were conducted using the dynamic light scattering (DLS) method with ZetaSizer Nano-ZS equipment at pH 7, a wavelength of 633 nm, a fixed scattering angle of 90°, and using a helium-neon (HeNe) polarising laser of 22 mW. The pore-volume, specific pore size, and surface area of CNCs were identified using the Brunauer–Emmett–Teller (BET) method using surface area and porosity analyzer (Model ASAP 2020, Micrometrics, Norcross, GA, USA) at a liquid nitrogen temperature of 77 K. Triplicate measurements were conducted, and the results were expressed as means ± standard error.

## 3. Results and Discussion

### 3.1. Morphological Analyses

**Figure 1:**
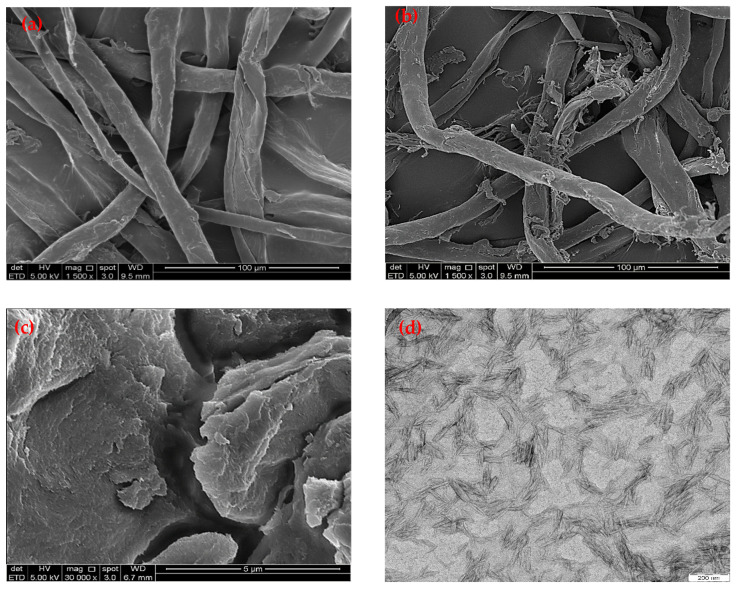

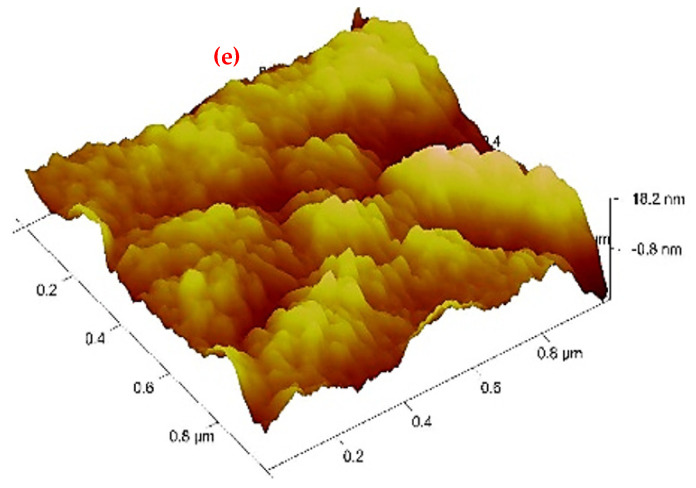
SEM image of waste cotton cloths (**a**), SEM image of scCO_2_ pulped fibre (**b**), SEM image of CNCs (**c**), TEM image of CNCs (**d**), and AFM image of CNCs (**e**).

Figure 1 displays the FE-SEM images of WCCs, scCO_2_ pulped fibre, and scCO_2_–CNCs. It was found that the surface of the WCCs (Figure 1a) was smooth. Moreover, the surface of the WCCs exhibited spiral fibers with a length of 104.50 ± 2.54 µm, width 30 ± 2.56 µm, and aspect ratio range of 4. However, the SEM image of the scCO_2_ pulped fiber shows that its surface was rough (Figure 1b). The outer layer of scCO_2_ pulped fiber was cracked and disrupted, indicating that the extraction lignin, hemicellulose, and lignin from the surface of the WCCs using the scCO_2_ [31,32]. The average length, width, and aspect ratio of the scCO_2_ pulped fiber was found to be 85.60 ± 1.73 µm, 13.60 ± 1.91 µm, and 6, respectively. The cellulose fiber shortened to a nano-size fiber after being treated with sulphuric acid hydrolysis, assisted with the ultrasonication process (Figure 1c). The sulphuric acid slashed the β-1,4-glycosidic bond of the cellulose repeating units (anhydroglucose) and penetrated the amorphous region of the cellulose, which was substantially broken down by the cellulose fiber and produced CNCs [33,34]. It was found that the sulphuric acid hydrolysis process successfully defibrillated the scCO_2_-treated WCCs’ fiber to CNCs of porous and rod-like structure. 

The energy-filtered transmission electron microscopy (TEM) was utilized to analyse the surface morphology of CNCs, as shown in Figure 1d. The length, width, and aspect ratio of CNCs were identified to be 100.03 ± 1.15 nm, 7.92 ± 0.53 nm and 13, respectively, as shown in Table 1. The surface morphology of CNCs was also observed using AFM, as shown in Figure 1e. It was found that the height and amplitude image of CNCs’ surface structure of the AFM micrograph supports the TEM image. The AFM image confirmed that CNCs had a smooth surface and small particle size. It can be seen in the amplitude image of the CNCs with a rod-like structure and a length less than 40.4 nm.

Wang et al. [22] utilized the acid hydrolysis process to fabricate CNCs from used cotton fabrics. The diameter of the CNCs was reported to be 3–35 nm, their length to be 28–470 nm, and their aspect ratio to be d 17 ± 15. Similarly, Huang et al. [21] utilized the acid hydrolysis of industrial textile waste to isolate CNCs for use as a filler in soy protein film. The width, length, and aspect ratios of CNCs were 11.18 ± 2.33 nm, 111.76 ± 38.73 nm, and 10 ± 3, respectively. It was found that the CNCs isolated in the present study had a lower length (100.03 ± 1.15 nm) and lower width (7.92 ± 0.53 nm) than those CNCs isolated from industrial textile waste [21] and old cotton cloths [22]. The scCO_2_ is a waterless cleaning technology and it does not alter the fiber quality due to its moderate pressure (7.4 MPa) and low operating temperature (31.1 °C) [15]. Therefore, the application of the scCO_2_ cleaning and pulping produce better quality CNCs from the WCCs. Studies reported that the porous material with the high surface area is viewed as an effective adsorbent in eliminating heavy metals from wastewater [29,35]. Hence, CNCs isolated from WCCs’ fiber could be utilized as an adsorbent to remove heavy metals.

### 3.2. ATR-FTIR Analysis

**Figure 2:**
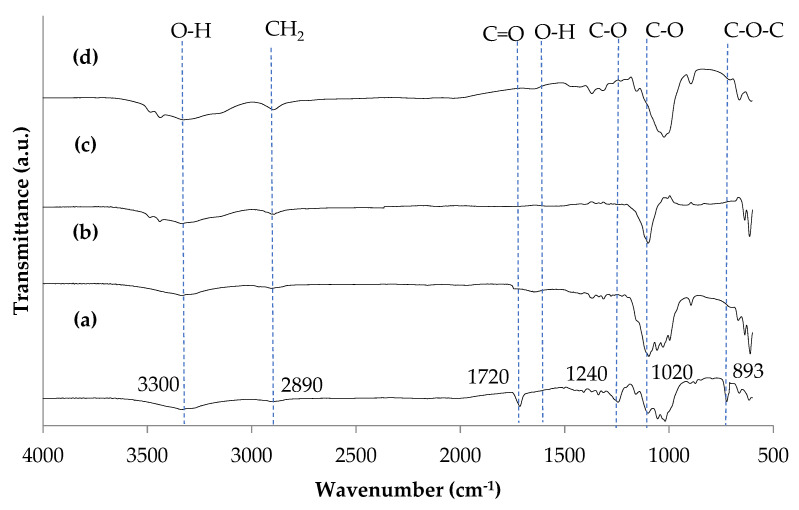
ATR-FTIR spectra of (**a**) waste cotton cloths, (**b**) scCO_2_ pulped fiber, (**c**) CNCs isolated from waste cotton cloths, and (**d**) CNCs after Cr(VI) adsorption.

The influence of the scCO_2_ pulping and sulphuric acid hydrolysis process for the production of CNCs from WCCs was determined by analyses of ATR-FTIR spectra of WCCs, scCO_2_ pulped fiber, and CNCs, as shown in Figure 2. It was observed that the peak obtained for WCCs, scCO_2_ pulped fiber, and CNCs were almost comparable, indicating that waste cotton cloths, scCO_2_ pulped fiber, and CNCs consist of the same chemical compositions. The broad peak obtained for the wavenumber 3300 cm^−1^ confirms the existence of the stretching vibration of O–H^−^ in WCCs, scCO_2_ pulped fiber, and CNCs. Additionally, the presence of the broad peak at wavenumber around 2890 cm^−1^ indicates the stretching vibration of the C–H bond (CH_2_) in WCCs, scCO_2_ pulped fiber, and CNCs. The peaks’ presence in WCCs spectra at a wavenumber of 1020 cm^−1^ and 1240 cm^−1^ represents the stretching vibration of C–O–C pyranose ring (antisymmetric in phase ring) and methoxyl-O-CH_3_, respectively. However, these peaks were not present in scCO_2_ pulped fiber and CNCs spectra, indicating that lignin was removed during scCO_2_ pulping [20,31]. Moreover, the peak at the wavenumber of 1720 cm^−1^ may be assigned to C=O bonds in carbonyl groups in hemicellulose and plane aromatic skeleton vibrations of lignin. However, the absence of the peak of 1720 cm^−1^ in scCO_2_ pulped fiber and CNCs spectra indicated that hemicellulose and lignin were removed from the WCCs during scCO_2_ pulping [19]. 

The peak appeared at 893 cm^−1^ in ATR-FTIR spectra of the WCCs, scCO_2_ pulped fiber, and CNCs can be espoused to the crystallinity index. It was found that the peak intensity at 893 cm^−1^ in ATR-FTIR spectra of the WCCs, scCO_2_ pulped fiber, and CNCs was increased gradually with scCO_2_ pulping and acid hydrolysis of WCCs [36]. This indicates that lignin, hemicellulose, and other extractives from WCCs were effectively eliminated as a result of the scCO_2_ pulping process. In our previous study, Mohamed et al. [1] isolated CNCs from WCCs using the conventional alkaline pulping, bleaching, and acid hydrolysis processes. It was found that alkaline pulping and bleaching of WCCs effectively removed lignin, hemicellulose, color, and other extractives from waste cotton cloths. Similarly, lignin, color, and other extractives were effectively removed from the WCCs with scCO_2_ pulping, indicating that the scCO_2_ could be utilized as an alternative to the conventional pulping and bleaching process of lignocellulose fiber processing. Figure 2d shows the FTIR spectra of CNCs after Cr(VI) adsorption. It was observed that the slight shifting of functional groups of C–O from 1020 cm^−1^ to 1000 cm^−1^, and of the O–H bond from 1720 cm^−1^ to 1640 cm^−1^ contributed to the bonding of Cr(VI) ions on the CNCs’ surface.

### 3.3. Zeta Potential Analyses

Zeta potential (ζ) is the potential difference between two layers (electrostatic and electric double-layer) of particles’ surfaces and the dispersant [37,38]. Generally, the zeta potential is one of the most important factors affecting the stability of colloidal, including causes of dispersion or aggregation. The zeta potential analysis was conducted to determine the surface charge of particles by measuring the magnitude attraction between particles or the magnitude of electrostatic charge repulsion [38]. The Zeta potential analysis of the CNCs found a negative surface charge (−32.6 mV) with a conductivity of 0.0283 mS/cm. Similarly, Metzer et al. [39] and Muneer et al. [40] reported that the CNCs fabricated with the sulphuric acid hydrolysis process had a negative zeta potential and negative surface charge. This is because of the forming of organosulfate (-OSO_3_^−^) groups on the surface of the cellulose during sulphuric acid hydrolysis of the cellulosic fiber [41]. However, the surface charge modification during the acid hydrolysis process increased the defibrillation of the cellulose because of the electrostatic repulsion between the similar negative surface charge [40]. Joseph and Singhvi [42] reported that a negative Zeta potential (between −30 mV to −40 mV) is considered a good stability of colloidal for dispersion due to a sufficient electrostatic repulsive force (i.e., repel each other) between individual particles. As a result, the CNCs would disperse uniformly and be electrostatically stable in an aqueous solution.

DLS is a widely used technology for determining particles’ size and size distribution [42,43]. It was observed that the particle size of the CNCs varied from 37.84 nm to 342 nm, with an average particle size of 109.5 nm. The size distribution curves of Maxwell–Boltzmann’s proved that 100% of CNCs’ particles size was in the nanometric range. However, the average particle size of CNCs obtained from DLS analyses slightly varied with the particles’ size determined by TEM analysis due to the non-homogeneous dispersion of CNCs in water [41]. Moreover, CNCs might be diffused across the aqueous medium in a vertical orientation against the light source, which later DLS has interpreted as a larger particle due to its slower diffusion speed, according to the Stoke–Einstein theory [44]. 

### 3.4. BET Analysis

**Figure 3:**
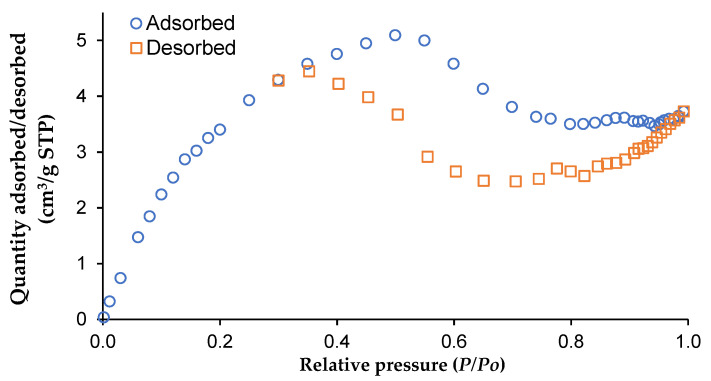
Nitrogen adsorption and desorption isotherm of CNCs using BET analysis.

The CNCs were analyzed using the BET method, nitrogen gas adsorption isotherms, and a surface area and porosity analyzer at a temperature of liquid nitrogen of 77 K, as shown in Figure 3. It was observed that the isotherm was fitted with the type IV adsorption isotherm, revealing that the isolated CNCs’ surface were mesoporous [45,46]. An average nitrogen gas area per molecule of 0.162 nm^2^ was used for predicting the pore size, pore volume, and specific surface area of CNCs. It was found that the amount of adsorbed gas on the surface of the CNCs particles was correlated to the specific surface area of the particles. The CNCs had a specific surface area of 26.12 m^2^/g with an average pore volume of 0.005 cm^3^/g and a pore width of 1.34 nm. Lu and Hsieh [45] determined the BET surface area of 13.36 m^2^/g of CNCs isolated from cotton cellulose. Abu–Danso et al. [46] reported that the BET surface area of CNCs to be about 8 m^2^/g. The high specific surface area of CNCs isolated from the WCCs was obtained in this present study might be due to the waterless pulping of the fiber. The scCO_2_ with the addition of ethanol as a co-solvent was extracted out of lignin, hemicellulose, color, and other extractives from the WCCs without deteriorating the fiber, which substantially increased the specific surface area of CNCs [47]. 

### 3.5. Adsorption of Cr(VI) Using CNCs

**Figure 4:**
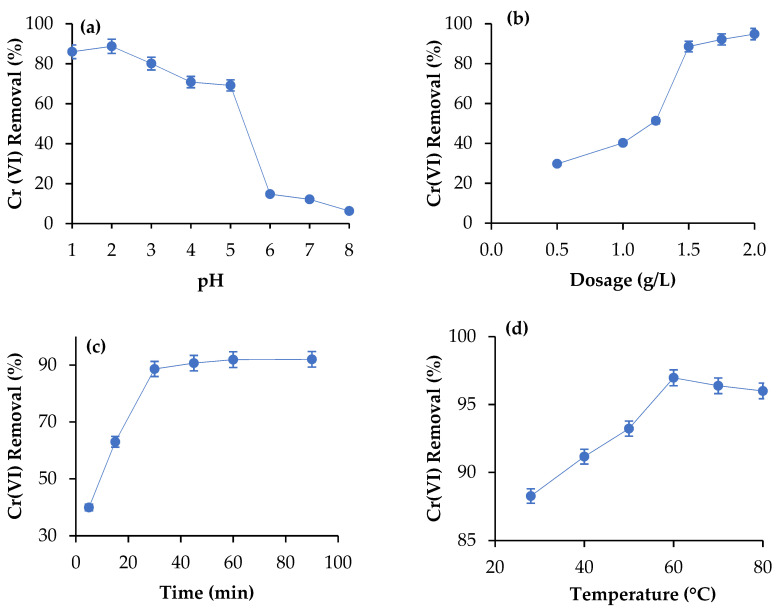
Removal of Cr(VI) from aqueous solution using CNCs isolated from waste cotton cloths: (**a**) effect of pH, (**b**) effect of doses (g/L), (**c**) effect of treatment time, and (**d**) effect of temperature (°C).

Figure 4 shows the influences of Cr(VI) removal using CNCs with varying pH, adsorbent doses, temperature, and treatment time. Figure 4a shows that the acidic pH strongly favoured Cr(VI) removal from pH 1 to pH 2 and decreased after that. The highest percentage Cr(VI) removal obtained was 88.72% at pH 2. It was found that percentage Cr(VI) removal decreased with the increase of pH from pH 2 to pH 8. pH is the crucial variable in metal ions’ separation using a bio-sorbent, where it influenced the adsorbent to adsorb the metal ions on the surface by modifying the particles’ surface charge [45]. Cr(VI) metal ions exist in aqueous solution as chromate (CrO_4_^2−^) and dichromate Cr_2_O_7_^2−^ [45,46]. At low pH (PH 1-PH 2), the percentage Cr(VI) removal was high due to abundant proton H^+^, and therefore CrO_4_^2−^ and Cr_2_O_7_^2−^ were protonated and adsorbed on the CNCs [46,47]. However, as the pH increased to over 2, the percentage of Cr(VI) removal decreased due to less proton H^+^ in aqueous solution to protonate Cr(VI) metal ions. Furthermore, the percentage of Cr(VI) removal was low at higher pH due to abundant OH^−^. Thus, it repels each other by electrostatic forces during adsorption and decreased percentage Cr(VI) removal. 

The effect of CNCs doses on the removal of Cr(VI) was determined with varying adsorbent doses 0.5 g/L to 2.0 g/L at pH 2, a treatment time of 30 min, at ambient temperature (28 ± 1 °C), and a Cr(VI) concentration of 100 ppm, as presented in Figure 4b. The percentage of Cr(VI) removal was increased with increasing CNCs doses from 0.5 g/L to 1.5 g/L and the increase of the Cr(VI) was negligible with further increasing of CNCs doses over 1.5 g/L. The increase of Cr(VI) removal with increasing CNCs can be attributed to the increase of negative surface charge available for Cr(VI) adsorption. The highest percentage Cr(VI) removal obtained was 88.59% at doses of 1.50 g/L. The percentage of Cr(VI) removal was constant over 1.50 g/L of CNCs doses due to the saturation of CNCs’ surface with the adsorbed Cr(VI) [46]. Moreover, the particles’ aggregation with a higher amount of CNCs led to the decreased affinity of adsorbing the Cr(VI) on the CNCs’ surface. Similarly, Wabaidur et al. [48] observed that the removal of Celestine blue and methylene blue increased with increasing doses of the silica-coated copper-decorated oxidized multi-walled carbon nanotubes as an adsorbent. 

The influence of treatment time on percentage Cr(VI) removal from aqueous solution using CNCs was determined with varying time from 5 min to 90 min at pH 2, doses of 1.50 g/L, an ambient temperature (28 ± 1 °C), and a Cr(VI) concentration of 100 ppm, as presented in Figure 4c. It was found that the highest percentage of Cr(VI) removal obtained was 88.64% at 30 min of treatment time. The percentage of Cr(VI) removal enhanced with the increase of treatment time from 5 min to 30 min; thereafter, the percentage Cr(VI) was negligible. However, the percentage of Cr(VI) removal increased with increasing treatment time due to the obtaining of the required contact time between CNCs’ surface and Cr(VI) for adsorption. Moreover, the increase of the active functional group with increasing adsorbent doses for adsorbing Cr(VI) increased the Cr(VI) removal [27,48]. Meanwhile, the percentage of Cr(VI) removal became negligible over 30 min treatment time because of the saturation of the CNCs’ surface with the adsorbed Cr(VI) [13].

The influence of temperature on the percentage of Cr(VI) removal from the aqueous solution g was determined by varying temperature from ambient temperature (28 ± 1 °C) to 80 °C, at pH 2, doses of 1.50 g/L, a Cr(VI) concentration of 100 ppm, and a treatment time of 30 min, as shown in Figure 4d. It was observed that the percentage Cr(VI) removal increased with the temperature increase from 28 ± 1 °C to 60 °C, and decreased thereafter. The highest percentage Cr(VI) removal obtained was 96% at 60 °C. The increase of Cr(VI) removal with increasing temperature from 28 ± 1 °C to 60 °C increased the kinetic energy, which enhanced the Cr(VI) binding on the surface of the CNCs and therefore increased the Cr(VI) adsorption. Moreover, an increase of the temperature decreased the solution’s viscosity, which substantially increased interaction between CNCs particles and Cr(VI) and therefore increased Cr(VI) adsorption [27,49]. However, the decrease of Cr(VI) removal with increasing temperature over 60 °C was due to the degrading of the CNCs particles, which substantially weakened the intermolecular force of CNCs particles and therefore decreased Cr(VI) binding on the surface of CNCs particles [13,26]. 

Kumari et al. [49] obtained about 97% of Cr(VI) removal using polyaniline-coated sugarcane bagasse composite as an adsorbent at pH 2.0, adsorbent doses of 3 g/L, temperature of 30 °C, and treatment time of 90 min. Lesaona et al. [50] found 98% of Cr(VI) adsorption using activated carbon as an adsorbent at pH 1, adsorbent doses 4g/L, and treatment time of 120 min. In the present study, about 97% of Cr(VI) removal was obtained using CNCs isolated from waste cotton cloths as an adsorbent at pH 2.0, at an ambient temperature, with adsorbent doses of 1.50 g/L, and at a treatment time of 30 min. Thus, it can be postulated that the CNCs isolated from waste cotton cloths using scCO_2_ pulping as an assisted sulphuric acid hydrolysis process has the potential to be used an effective adsorbent for the Cr(VI) removal. 

### 3.6. Adsorption Equilibrium Studies

The Freundlich and Langmuir isothermal models were employed to illustrate the Cr(VI) removal behavior using CNCs isolated from WCCs, as shown in Supplementary Figure S1. The Langmuir isotherm model illustrates that the adsorption of heavy metals ion occurred on the adsorbent in monolayer formation and in homogeneous distribution [51,52,53,54]. The Langmuir isotherm model for Cr(VI) removal using CNCs isolated from the WCCs as an adsorbent is displayed in Figure S1a. The Langmuir constant (*a*, L/mg) and the maximum adsorption values (*b*, mg/mg) were determined to be 0.422 L/mg, and 0.082 mg/mg, respectively. The Freundlich isotherm model for the removal of Cr(VI) using CNCs as an adsorbent is shown in Supplementary Figure S1b. The Freundlich exponential constant (*n*) and Freundlich affinity constant values (*k_f_*, L/mg) for Cr(VI) removal were 5.862 and 0.248 L/mg, respectively.

Table 2 shows the coefficient of determination (*R*^2^) values of the Freundlich and Langmuir isotherm model for the removal of Cr(VI) using CNCs as an adsorbent. The *R*^2^ values of Freundlich and Langmuir isotherm model were 0.954 and 0.931, respectively. The greater *R*^2^ values of the Freundlich isotherm model than those of the Langmuir isotherm model reveal that the Freundlich isotherm model was the best-fitted isotherm model to describe the Cr(VI) removal behavior using CNCs as an adsorbent. Thus, it can be postulated that Cr(VI) adsorption occurred on the surface of CNCs in a multi-layer formation and in non-uniform distributions [54]. 

### 3.7. Adsorption Kinetics

The adsorption kinetics for Cr(VI) removal depends on physicochemical characteristics of CNCs and mass transfer, as shown in Figure S2. However, the Cr(VI) removal rate was determined by fitting the experimental data with the kinetic model equations. The adsorption kinetics parameters, such as *R*^2^, *k*_1_, *k*_2_, and *q_e_* values, were determined from the slop and intercept of plots obtained for pseudo-first-order and pseudo-second-order kinetic models, as presented in Table 3. It was observed that *q_e_* (experimental) values merely increased with the increase of temperature from 28 to 60 °C, while it decreased at 80 °C. The increase of the *q_e_* value with increasing temperature can be related to the increase of the collision between the Cr(VI) ions and CNCs particles, which enhance interaction between Cr(VI) and CNCs particles and therefore increase the Cr(VI) removal. However, the decrease of the Cr(VI) uptake over 60 °C might be attributed to weaker bonding of Cr(VI) ions on the CNCs’ surface due to the high temperature, which led to the Cr(VI) detaching/escaping from the CNCs surface and therefore a decrease in the adsorption efficiency [48]. The highest Cr(VI) removal obtained was 0.065 mg/mg at a temperature of 60 °C, a pH of 2, with adsorbent doses of 1.5 g/L, and a concentration of 100 ppm. Zeng et al. [49] reported the maximum Cr(VI) uptake of 0.022 mg/mg using chitosan/CNCs grafted with carbon dots composite hydrogel. However, the higher Cr(VI) removal obtained in the present study was due to the high porosity and large surface area of the isolated CNCs from WCCs. 

The correlation coefficient (*R*^2^) and variations between theoretical and experimental *q_e_* values were employed to predict the best-described kinetics model. It was found that the *R*^2^ values were higher for the pseudo-second-order kinetic model than the *R*^2^ values for the pseudo-first-order kinetic model. Moreover, the theoretical *q_e_* values were closely matched to the pseudo-second-order kinetics model. Thus, it can be postulated that the pseudo-second-order kinetic model was the better kinetic model for describing the adsorption kinetics for the removal of Cr(VI) using CNCs as an adsorbent.

### 3.8. Adsorption Thermodynamics

Thermodynamics parameters such as Δ*H°*, Δ*G°*, and Δ*S°* were determined for Cr(VI) removal using CNCs as an adsorbent, as shown in Figure S3. Table 4 shows the Δ*S°*, Δ*H°* and Δ*G°* values for Cr(VI) removal using CNCs as an adsorbent. It was found that the Δ*G°* value was negative for Cr(VI) removal from −0.395 to −5.033 kJ/mol, indicating that the adsorption process was favourable and spontaneous. The Δ*G°* value is an indicator to determine whether the adsorption process is physisorption (−20 to 0 kJ/mol) or chemisorption (−80 to −400 kJ/mol) [53]. The negative Δ*G°* values obtained were in the range of the physisorption process (−20 to 0 kJ/mol), indicating that the removal of Cr(VI) occurred as a result of the porous CNCs’ surface or by the binding of the Cr(VI) by the active functional groups (i.e., –OH, –SO_3_) present on the CNCs’ surface. It was found that Δ*G°* decreased with the increase of temperature from 28 to 60 °C, indicating that the external energy source influenced adsorption efficiency. The Δ*H°* and Δ*S°* values for Cr(VI) removal were determined to be 27.407 kJ/mol and 0.092 kJ/mol.K, respectively. The positive Δ*H°* value reveals that the Cr(VI) adsorption on the surface of CNCs was endothermic [54]. Meanwhile, the positive Δ*S°* value indicates that the removal of Cr(VI) from aqueous solution using CNCs increased randomness [54,55]. The thermodynamic behavior of the Cr(VI) removal obtained in the present study are in agreement with studies reported by Zhang et al. [55] and Park et al. [56]. For instance, Park et al. [56] reported that the adsorption of Cr(VI) using poly(acryloyl hydrazide) grafted CNCs was spontaneous and endothermic.

## 4. Conclusions

In this study, CNCs isolated from scCO_2_-treated WCCs were utilized as an adsorbent for Cr(VI) removal from aqueous solution. The morphological analyses showed that scCO_2_-CNCs had a porous surface, and a crystalline and rod-like structure, with a length of 100.03 ± 1.15 nm and a width of 7.92 ± 0.53 nm, respectively. ATR-FTIR analysis showed that scCO_2_ effectively removed lignin, hemicellulose, color, and extractives from waste cotton cloths. Zeta potential and BET analyses revealed that the CNCs isolated from the WCCs were negative surface charged with a specific surface area of 26.12 m^2^/g. The adsorption of Cr(VI) using the isolated CNCs from WCCs revealed that the maximum Cr(VI) removal obtained was 96.97% at pH 2, using 1.5 g/L of CNCs, at a temperature of 60 °C, and with a treatment time of 30 min. Adsorption equilibrium studies showed that the removal of Cr(VI) occurred in multilayer formation and in non-uniform distributions. The pseudo-second-order kinetic model was the best-fitted models for describing Cr(VI) adsorption behavior. The thermodynamic properties analyses showed that the removal of Cr(VI) using CNCs as an adsorbent was spontaneous and endothermic. The findings of the present study reveal that the CNCs isolated from the scCO_2_-treated WCCs could be utilized as an effective adsorbent for the removal of trace elements from wastewater. Moreover, the isolation of CNCs from the WCCs-assisted scCO_2_ pulping would enhance sustainable utilization of waste materials while minimizing wastewater generation and environmental pollution. 

## Figures and Tables

**Table 1:** Particle size of waste cotton cloths, scCO_2_ pulped fibre, and CNCs.

	Waste Cotton Cloths	scCO_2_ Pulped Fibre	CNCs
Length	104.50 ± 2.54 µm	85.60 ± 1.73 µm	100.03 ± 1.15 nm
Width	30 ± 2.56 µm	13.60 ± 1.91 µm	7.92 ± 0.53 nm
Aspect ratio	4	6	13

**Table 2:** Adsorption isotherm modeling for the removal of Cr(VI) from aqueous solution using CNCs as an adsorbent.

Langmuir Isotherm			Freundlich Isotherm		
*R* ^2^	*a* (L/mg)	*b* (mg/mg)	*R* ^2^	*k_f_* (L/mg)	*n*
0.931	0.422	0.082	0.954	0.248	5.862

**Table 3:** Adsorption kinetics modeling for the removal of Cr(VI) from aqueous solution using CNCs as an adsorbent.

Temperature (°C)	*q_e_* (exp.) (mg/mg)	Pseudo-First-Order Kinetics			Pseudo-Second-Order Kinetics		
		*q_e_* (mg/mg)	*k*_1_ (1/min)	*R* ^2^	*q_e_* (mg/mg)	*k*_2_ (mg/mg.min)	*R* ^2^
28	0.0613	0.2018	0.1409	0.9615	0.0785	0.898	0.9751
40	0.0627	0.1250	0.1344	0.9916	0.0730	1.649	0.9910
60	0.0655	0.0546	0.1177	0.9784	0.0709	3.391	0.9977
80	0.0648	0.0354	0.1232	0.9899	0.0674	7.297	0.9960

**Table 4:** Adsorption thermodynamics for the removal of Cr(VI) from aqueous solution using CNCs as an adsorbent.

Temperature (°C)	Δ*G*° (kJ/mol)	Δ*H*° (kJ/mol)	Δ*S*° (kJ/mol.K)
28	−0.395	27.407	0.092
40	−1.201		
60	−3.434		
80	−5.033		

## Data Availability

Not Applicable.

## Supplementary Materials

The following are available online at https://www.mdpi.com/article/10.3390/polym14050887/s1, Figure S1: Adsorption isotherm modeling for the removal of Cr(VI) from aqueous solution using CNCs adsorbent.(a) Langmuir isotherm, and (b) Freundlich isotherm. Experimental condition: pH 2, doses of 1.5 g/L, and concentration of 100 ppm; Figure S2: Adsorption kinetics modeling for the removal of Cr(VI) from aqueous solution using CNCs adsorbent.(a) pseudo-first-order kinetic model, and (b) pseudo-second-order kinetic model. Experimental condition: pH 2, doses of 1.5 g/L, and concentration of 100 ppm; Figure S3: Adsorption thermodynamics for the removal of Cr(VI) from aqueous solution using CNCs adsorbent. Experimental condition: pH 2, doses of 1.5 g/L, initial concentration of 100 ppm, and 30 min.

## References

[1] Mohamed S.H., Hossain M.S., Mohamad Kassim M.H., Ahmad M.I., Omar F.M., Balakrishnan V., Zulkifli M., Yahaya A.N.A. (2021). Recycling WCCs for the Isolation of Cellulose Nanocrystals: A Sustainable Approach. Polymers.

[2] Yousef S., Tatariants M., Tichonovas M., Sarwar Z., Jonuškienė I., Kliucininkas L. (2019). A new strategy for using textile waste as a sustainable source of recovered cotton. Res. Conserv. Recycl..

[3] Patti A., Cicala G., Acierno D. (2021). Eco-Sustainability of the Textile Production: Waste Recovery and Current Recycling in the Composites World. Polymers.

[4] Abdel-Shafy H.I., Mansour M.S.M. (2018). Solid waste issue: Sources, composition, disposal, recycling, and valorization. Egypt. J. Petrol..

[5] Zheng W., Phoungthong K., Lü F., Shao L.-M., He P.-J. (2013). Evaluation of a classification method for biodegradable solid wastes using anaerobic degradation parameters. Waste Manag..

[6] Zhang H., Chen Y., Wang S., Ma L., Yu Y., Dai H., Zhang Y. (2020). Extraction and comparison of cellulose nanocrystals from lemon (Citrus limon) seeds using sulfuric acid hydrolysis and oxidation methods. Carbohydr. Polym..

[7] Verma C., Chhajed M., Gupta P., Roy S., Maji P.K. (2021). Isolation of cellulose nanocrystals from different waste bio-mass collating their liquid crystal ordering with morphological exploration. Int. J. Biol. Macromol..

[8] Huang C., Yu H., Abdalkarim S.Y.H., Li Y., Chen X., Yang X., Zhou Y., Zhang L. (2022). A comprehensive investigation on cellulose nanocrystals with different crystal structures from cotton via an efficient route. Carbohydr. Polym..

[9] Stark N.M., Matuana L.M. (2021). Trends in sustainable biobased packaging materials: A mini review. Mater. Today Sustain..

[10] Yue L., Amirkhosravi M., Ke K., Gray T.G., Manas-Zloczower I. (2021). Cellulose Nanocrystals: Accelerator and Reinforcing Filler for Epoxy Vitrimerization. ACS Appl. Mater. Interfaces.

[11] Rivers G., Yu L., Zhao B. (2019). Cellulose Nanocrystal and Silver Nanobelt Gel: Cooperative Interactions Enabling Dispersion, Colloidal Gels, and Flexible Electronics. Langmuir.

[12] Ganguly K., Patel D.K., Dutta S.D., Shin W.-C., Lim K.-T. (2020). Stimuli-responsive self-assembly of cellulose nanocrystals (CNCs): Structures, functions, and biomedical applications. Int. J. Biol. Macromol..

[13] Li W., Ju B., Zhang S. (2019). Preparation of cysteamine-modified cellulose nanocrystal adsorbent for removal of mercury ions from aqueous solutions. Cellulose.

[14] Ilias M.K.M., Balakrishnan V., Zuknik M.H., Al-Gheethi A., Ghfar A.A., Hossain M.S. (2021). Supercritical CO_2_ separation of lipids from chicken by-product waste for biodiesel production: Optimization, kinetics, and thermodynamics modeling. Biomass Convers. Biorefinery.

[15] Allafi F.A., Hossain M.S., Shaah M., Lalung J., Ab Kadir M.O., Ahmad M.I. (2021). Waterless sterilization and cleaning of sheep wool fiber using supercritical carbon dioxide. Text. Res. J..

[16] Easmin M.S., Sarker M.Z.I., Ferdosh S., Shamsudin S.H., Yunus K.B., Uddin M.S., Sarker M.M.R., Akanda M.J.H., Hossain M.S., Khalil H.A. (2015). Bioactive compounds and advanced processing technology: *Phaleria macrocarpa* (sheff.) Boerl, a review. J. Chem. Technol. Biotechnol..

[17] Gustinelli G., Eliasson L., Svelander C., Alminger M., Ahrné L. (2018). Supercritical CO_2_ extraction of bilberry (*Vaccinium myrtillus* L.) seed oil: Fatty acid composition and antioxidant activity. J. Supercrit. Fluids.

[18] Omar A.K.M., Tengku Norsalwani T.L., Asmah M.S., Badrulhisham Z.Y., Easa A.M., Omar F.M., Hossain M.S., Zuknik M.H., Nik Norulaini N.A. (2018). Implementation of the supercritical carbon dioxide technology in oil palm fresh fruits bunch sterilization: A review. J. CO2 Util..

[19] Pasquini D., Pimenta M.T.B., Ferreira L.H., Curvelo A.A.S. (2005). Sugar cane bagasse pulping using supercritical CO_2_ associated with co-solvent 1-butanol/water. J. Supercrit. Fluids.

[20] Vedoya C.I., Vallejos M.E., Area M.C., Felissia F.E., Raffaeli N., da Silva Curvelo A.A. (2020). Hydrothermal treatment and organosolv pulping of softwood assisted by carbon dioxide. Ind. Crops Prod..

[21] Huang S., Liu X., Chang C., Wang Y. (2020). Recent developments and prospective food-related applications of cellulose nanocrystals: A review. Cellulose.

[22] Wang Z., Yao Z., Zhou J., Zhang Y. (2017). Reuse of waste cotton cloth for the extraction of cellulose nanocrystals. Carbohydr. Polym..

[23] Maciel M.M.Á.D., Benini K.C.C.d.C., Voorwald H.J.C., Cioffi M.O.H. (2019). Obtainment and characterization of nanocellulose from an unwoven industrial textile cotton waste: Effect of acid hydrolysis conditions. Int. J. Biol. Macromol..

[24] Jobby R., Jha P., Yadav A.K., Desai N. (2018). Biosorption and biotransformation of hexavalent chromium [Cr(VI)]: A comprehensive review. Chemosphere.

[25] Vaiopoulou E., Gikas P. (2020). Regulations for chromium emissions to the aquatic environment in Europe and elsewhere. Chemosphere.

[26] Choudhary B., Paul D. (2018). Isotherms, kinetics and thermodynamics of hexavalent chromium removal using biochar. J. Environ. Chem. Eng..

[27] Billah R.E.K., Khan M.A., Park Y.-K., AM A., Majdoubi H., Haddaji Y., Jeon B.-H. (2021). A Comparative Study on Hexavalent Chromium Adsorption onto Chitosan and Chitosan-Based Composites. Polymers.

[28] Ali Khan M., Govindasamy R., Ahmad A., Siddiqui M.R., Alshareef S.A., Hakami A.A.H., Rafatullah M. (2021). Carbon Based Polymeric Nanocomposites for Dye Adsorption: Synthesis, Characterization, and Application. Polymers.

[29] Liu C., Jin R.-N., Ouyang X.-K., Wang Y.-G. (2017). Adsorption behavior of carboxylated cellulose nanocrystal—Polyethyleneimine composite for removal of Cr(VI) ions. Appl. Surface Sci..

[30] Hossain M.S., Nik Ab Rahman N.N., Balakrishnan V.F.M., Alkarkhi A., Ahmad Rajion Z., Ab Kadir M.O. (2015). Optimizing supercritical carbon dioxide in the inactivation of bacteria in clinical solid waste by using response surface methodology. Waste Manag..

[31] Lyu P., Zhang Y., Wang X., Hurren C. (2021). Degumming methods for bast fibers—A mini review. Ind. Crops Prod..

[32] Surup G.R., Hunt A.J., Attard T., Budarin V.L., Forsberg F., Arshadi M., Abdelsayed V., Shekhawat D., Trubetskaya A. (2020). The effect of wood composition and supercritical CO_2_ extraction on charcoal production in ferroalloy industries. Energy.

[33] Fatah I.Y.A., Khalil H.P.S.A., Hossain M.S., Aziz A.A., Davoudpour Y., Dungani R., Bhat A. (2014). Exploration of a Chemo-Mechanical Technique for the Isolation of Nanofibrillated Cellulosic Fiber from Oil Palm Empty Fruit Bunch as a Reinforcing Agent in Composites Materials. Polymers.

[34] Razali N., Hossain M.S., Taiwo O.A., Ibrahim M., Mohd Nadzri N.W., Razak N., Mohammad Rawi N.F., Mohd Mahadar M., Mohamad Kassim M.H. (2017). Influence of Acid Hydrolysis Reaction Time on the Isolation of Cellulose Nanowhiskers from Oil Palm Empty Fruit Bunch Microcrystalline Cellulose. BioResources.

[35] Mohiuddin G., Ghosh S., Begum N., Debnath S., Turlapati S., Rao D.S.S., Nandiraju R.V.S. (2018). Amide linkage in novel three-ring bent-core molecular assemblies: Polar mesophases and importance of H-bonding. Liq. Cryst..

[36] Thambiraj S., Ravi Shankaran D. (2017). Preparation and physicochemical characterization of cellulose nanocrystals from industrial waste cotton. Appl. Surface Sci..

[37] Derkani M.H., Fletcher A.J., Fedorov M., Abdallah W., Sauerer B., Anderson J., Zhang Z.J. (2019). Mechanisms of surface charge modification of carbonates in aqueous electrolyte solutions. Colloids Interfaces.

[38] Dhali K., Ghasemlou M., Daver F., Cass P., Adhikari B. (2021). A review of nanocellulose as a new material towards environmental sustainability. Sci. Total Environ..

[39] Metzger C., Auber D., Dahnhardt-Pfeiffer S., Briesen H. (2020). Agglomeration of cellulose nanocrystals: The effect of secondary sulfates and their use in product separation. Cellulose.

[40] Muneer R., Hashmet M.R., Pourafshary P. (2020). Fine migration control in sandstones: Surface force analysis and application of DLVO theory. ACS Omega.

[41] Jordan J.H., Easson M.W., Condon B.D. (2020). Cellulose hydrolysis using ionic liquids and inorganic acids under dilute conditions: Morphological comparison of nanocellulose. RSC Adv..

[42] Joseph E., Singhvi G. (2019). Multifunctional nanaocrystals for cancer therapy: A potential nanocarrier. Nanomater. Drug Deliv. Ther..

[43] Morantes D., Munoz E., Kam D., Shoseyov O. (2019). Highly charged cellulose nanocrystals applied as a water treatment flocculant. Nanomaterials.

[44] Stetefeld J., McKenna S.A., Patel T.R. (2016). Dynamic light scattering: A practical guide and applications in biomedical sciences. Biophys. Rev..

[45] Lu P., Hsieh Y.-L. (2010). Preparation and properties of cellulose nanocrystals: Rods, spheres, and network. Carbohydr. Polym..

[46] Abu-Danso E., Srivastava V., Sillanpää M., Bhatnagar A. (2017). Pretreatment assisted synthesis and characterization of cellulose nanocrystals and cellulose nanofibers from absorbent cotton. Int. J. Biol. Macromol..

[47] Escobar E.L.N., da Silva T.A., Pirich C.L., Corazza M.L., Pereira Ramos L. (2020). Supercritical Fluids: A Promising Technique for Biomass Pretreatment and Fractionation. Front. Bioeng. Biotechnol..

[48] Wabaidur S.M., Khan M.A., Siddiqui M.R., Otero M., Jeon B.-H., Alothman Z.A., Hakami A.A.H. (2020). Oxygenated functionalities enriched MWCNTs decorated with silica coated spinel ferrite—A nanocomposite for potentially rapid and efficient de-colorization of aquatic environment. J. Mol. Liq..

[49] Kumari B., Tiwary R.K., Yadav M. (2022). Non linear regression analysis and response surface modeling for Cr (VI) removal from aqueous solution using poly-aniline coated sugarcane bagasse (PANI@SB) composites as an adsorbent. Surfaces Interfaces.

[50] Lesaoana M., Mlaba R.P.V., Mtunzi F.M., Klink M.J., Ejidike P., Pakade V.E. (2019). Influence of inorganic acid modification on Cr(VI) adsorption performance and the physicochemical properties of activated carbon. S. Afr. J. Chem. Eng..

[51] Zaime M.Z.A., Sarjadi M.S., Rahman M.L. (2021). Heavy metals removal from water by efficient adsorbents. Water.

[52] Alvarez-Ayuso E., Garcia-Sanchez A., Querol X. (2007). Adsorption of Cr(VI) from synthetic solutions and electroplating wastewaters on amorphous aluminium oxide. J. Hazard. Mater..

[53] Khalid A.M., Hossain M.S., Ismail N., Khalil N.A., Balakrishnan V., Zulkifli M., Yahaya A.N.A. (2021). Isolation and Characterization of Magnetic Oil Palm Empty Fruits Bunch Cellulose Nanofiber Composite as a Bio-Sorbent for Cu(II) and Cr(VI) Removal. Polymers.

[54] Mat Yasin N.M.F., Hossain M.S., Abdul Khalil H.P.S., Zulkifli M., Al-Geethi A., Asis A.J., Ahmad Yahaya A.N. (2020). Treatment of palm oil refinery effluent using tannin as a polymeric coagulant: Isotherm, kinetics, and thermodynamics analyses. Polymers.

[55] Zhang H., Xiao R., Li R., Ali A., Chen A., Zhang Z. (2020). Enhanced aqueous Cr(VI) removal using chitosan-modified magnetic biochars derived from bamboo residues. Chemosphere.

[56] Park S.-H., Shin S.S., Park C.H., Jeon S., Gwon J., Lee S.-Y., Kim S.-J., Kim H.-J., Lee J.-H. (2020). Poly(acryloyl hydrazide)-grafted cellulose nanocrystal adsorbents with an excellent Cr(VI) adsorption capacity. J. Hazard. Mater..

