# Supplementary material for: Biosorption of Cr(VI) Using Cellulose Nanocrystals Isolated from the Waterless Pulping of Waste Cotton Cloths with Supercritical CO2: Isothermal, Kinetics, and Thermodynamics Studies
Source: Polymers (Basel). 2022 Feb 23;14(5):887. doi: 10.3390/polym14050887 (PMC8912417; doi:10.3390/polym14050887)
Supplement: Supplementary file 1 [file polymers-14-00887-s001.zip › polymers-1596753-supplementary.pdf]

Supplementary Data

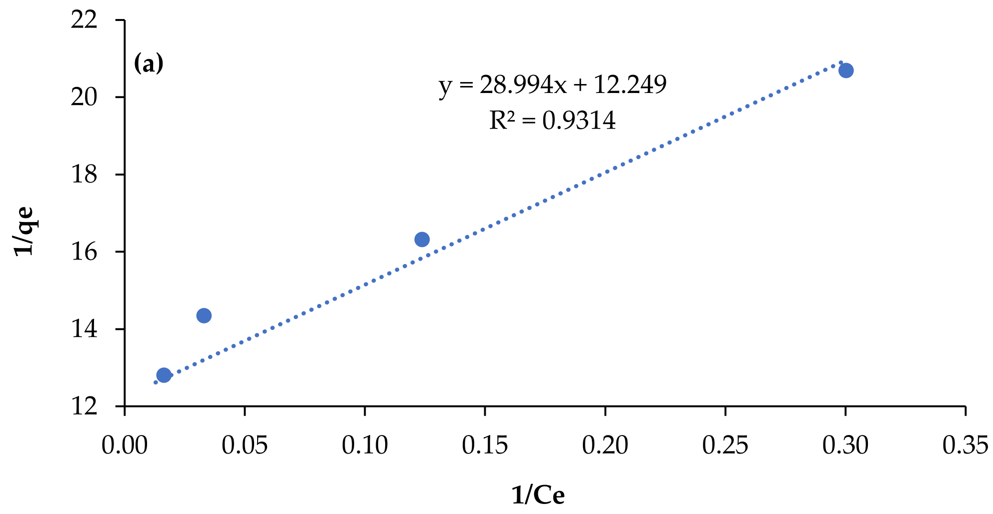

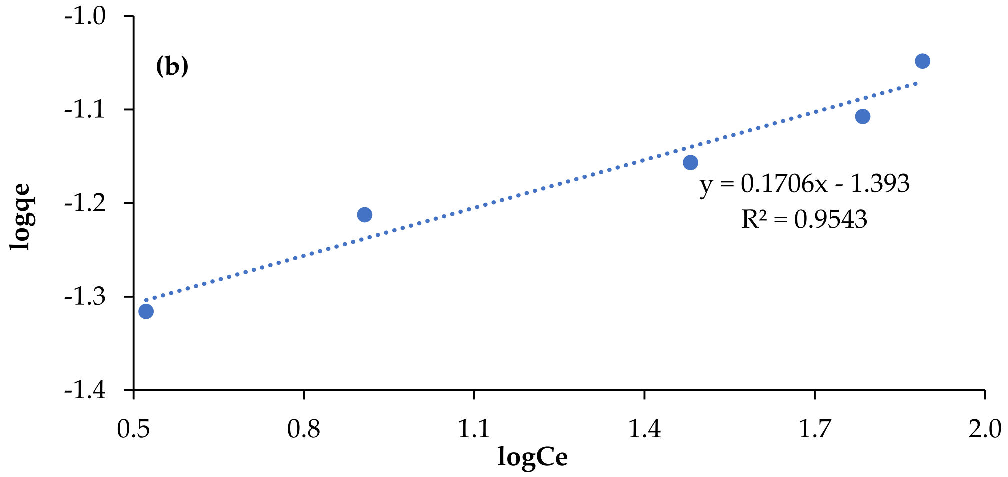

Figure S1. Adsorption isotherm modeling for the removal of Cr(VI) from aqueous solution using CNCs adsorbent. (a) Langmuir isotherm, and (b) Freundlich isotherm. Experimental condition: pH 2, doses of 1.5 g/L, and concentration of 100 ppm.

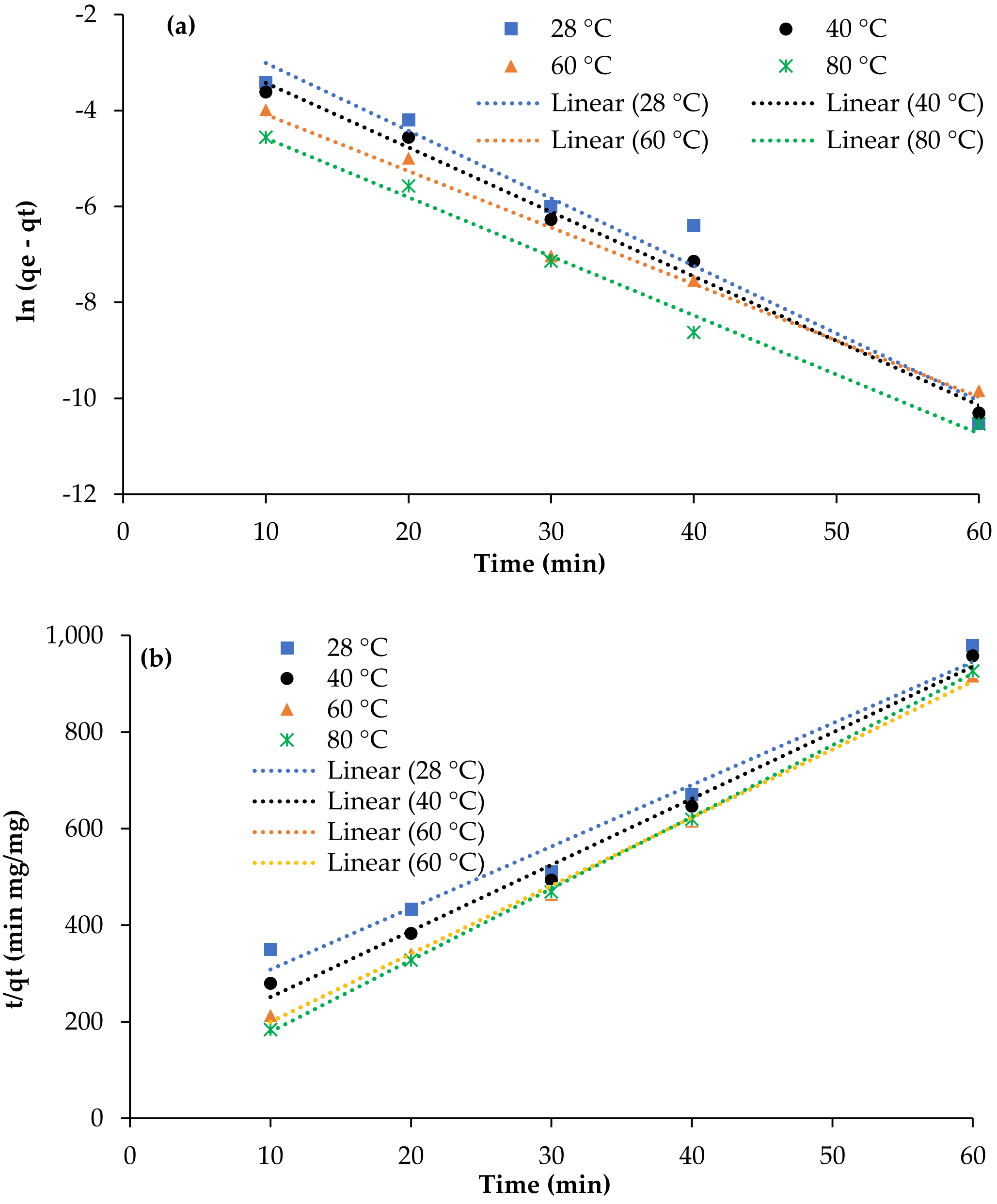

Figure S2. Adsorption kinetics modeling for the removal of Cr(VI) from aqueous solution using CNCs adsorbent. (a) pseudo-first-order kinetic model, and (b) pseudo-second-order kinetic model. Experimental condition: pH 2, doses of 1.5 g/L, and concentration of 100 ppm.

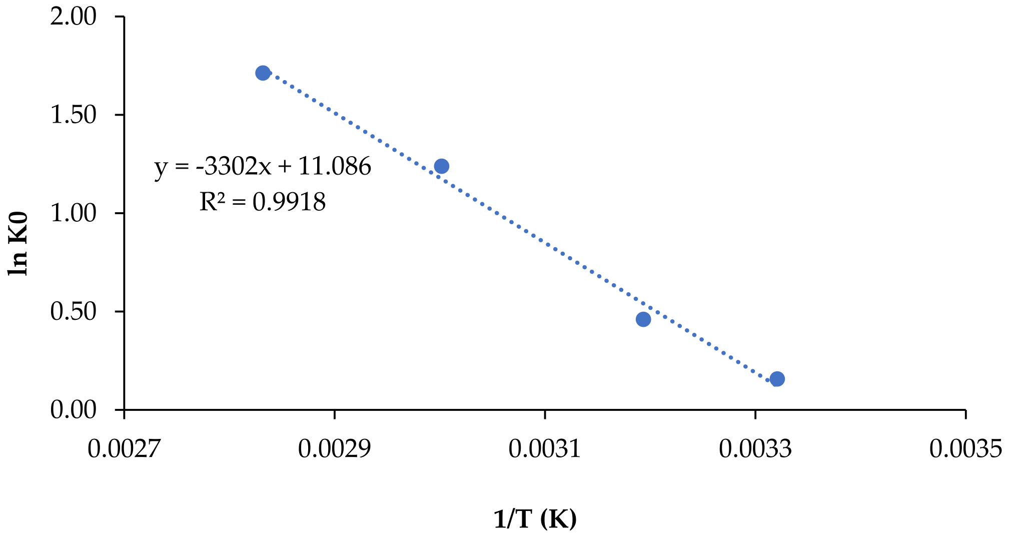

Figure S3. Adsorption thermodynamics for the removal of Cr(VI) from aqueous solution using CNCs adsorbent. Experimental condition: pH 2, doses of 1.5 g/L, initial concentration of 100 ppm, and 30 min.
